# Association of Impulse Control Disorders with Cognitive Performance and Frontal Dysfunction in Patients with Parkinson’s Disease

**DOI:** 10.3390/jcm15051698

**Published:** 2026-02-24

**Authors:** Maria Bougia, Aristeidis Papadaniil, Evangelia Smaragdaki, Nikolaos Papagiannakis, Athina-Maria Simitsi, Ion Beratis, Dionysia Kontaxopoulou, Stella Fragkiadaki, Ioanna Alefanti, Evangelos Sfikas, Ioanna Alexandratou, Roubina Antonelou, Sokratis G. Papageorgiou, Leonidas Stefanis, Christos Koros

**Affiliations:** 1 1st Department of Neurology, Eginition Hospital, Medical School, National and Kapodistrian University of Athens, 11528 Athens, Greece; mariabougia97@gmail.com (M.B.); apapadaniel@gmail.com (A.P.); eua_smaragdaki@hotmail.com (E.S.); nikolas.papagia@gmail.com (N.P.); simitsh@yahoo.gr (A.-M.S.); d.kontaxopoulou@hotmail.com (D.K.); st.fragkiadaki@gmail.com (S.F.); jalefanti@gmail.com (I.A.); vasfikas@gmail.com (E.S.); io.alexandratou@gmail.com (I.A.); rantonelou@gmail.com (R.A.); sokpapa@med.uoa.gr (S.G.P.); lstefanis@bioacademy.gr (L.S.); 2 Psychology Department, American College of Greece, Deree, 6 Gravias Street, 15342 Athens, Greece; ionas96@hotmail.com

**Keywords:** Parkinson’s disease, frontal lobe, impulsive compulsive disorders, cognitive status

## Abstract

**Background**: Frontal lobe circuit dysfunction, including the mesolimbic network, plays an important role in learning reward behaviors and is involved in the development of impulsive compulsive disorders (ICDs) in Parkinson’s disease (PD). ICDs in PD are characterized by disinhibited, reward-driven behaviors performed with poor impulse control, often linked to dopaminergic treatment. The purpose of the present study was to assess the presence of these behaviors in relation to frontal dysfunction and overall cognitive status in a cohort of patients with sporadic PD. **Methods**: The study consisted of 55 patients (*n* = 55), 36 males (65.5%), diagnosed with Parkinson’s disease, assessed at the First Neurological Clinic of Eginition University Hospital in Athens. The participants had a mean age of 62.6 (±13.54) years, with an average of 11.94 (±3.00) years of education and a mean disease duration of 7.17 (±5.90) years. The evaluation tools used to assess the participants were the Questionnaire for Impulsive-Compulsive Disorders in Parkinson’s Disease (QUIP), Montreal Cognitive Assessment (MoCA), Frontal Assessment Battery (FAB), and Geriatric Depression Scale (GDS). **Results**: The mean score on the QUIP was 0.64 (±1.05), with a threshold of 1. Of the total number of patients (*n* = 55), 18 (32.72%) showed behaviors related to ICDs. The most commonly reported impulsive compulsive behavior was an excessive preoccupation with hobbies (*n* = 7, 38.9%), followed by a tendency toward gambling (*n* = 6, 33.3%). The mean score on the MoCA scale was 24.69/30 (±4.25), while the mean score on the FAB scale was 14.70/18 (±2.45). Pearson’s correlation analysis revealed a moderate positive correlation between total MoCA score and FAB (*r* = 0.588, *p* < 0.000) and a weak to moderate negative correlation between MoCA score and QUIP (*r* = −0.291, *p* = 0.038). Additionally, there was a statistically significant negative correlation between QUIP scores and performance on the MoCA attention subtests (Forward Digit Span, Backward Digit Span, and Vigilance tasks), (*r* = −0.389, *p* = 0.009). **Conclusions**: Lower global cognitive function, as measured by the MoCA, was strongly associated with reduced frontal lobe function, as measured by the FAB, in Parkinson’s patients. Additionally, lower scores on the MoCA, particularly in the attention subtests, showed a weak to moderate correlation with increased impulsive compulsive behaviors, as measured by the QUIP.

## 1. Introduction

Parkinson’s disease (PD) is a progressive neurodegenerative disorder characterized primarily by motor symptoms such as rest tremors, bradykinesia, and muscle rigidity [1]. However, increasing research and clinical interest are focusing on the non-motor spectrum of the disease, which includes a set of heterogeneous symptoms such as cognitive impairment, mood disorders (depression, anxiety), psychotic symptomatology, and impulse control disorders (ICDs). These manifestations, although historically underreported, have now been shown to profoundly affect the daily functioning, treatment engagement, and overall quality of life of patients and are associated with increased caregiver burden rates and increased health service utilization [2,3,4]. At the same time, non-motor manifestations may temporally precede motor symptoms and may escape early diagnosis, thus worsening the clinical prognosis.

In accordance with numerous meta-analyses covering the period 2000–2017, such as that of Martini et al. (2018) [5], ICDs are detected with high frequency in patients with Parkinson’s disease. Reported rates range from 6% to 15% for individual behaviors such as compulsive gambling, hypersexuality, compulsive buying, and hyperphagic episodes, especially in patients under chronic treatment with dopaminergic agonists. These agonists (including ropinirole and pramipexole) have been found to increase the relative risk of ICDs by 2.5 to 3 times compared to other drug interventions, as documented in recent studies [6]. Notably, the pharmacological association, although strong, is insufficient to explain the occurrence of ICDs, as evident from the meta-analysis by Santangelo et al. (2017) [3] and the review by Carbone and Djamshidian (2024) [7]. These studies focus on the role of executive functions, particularly cognitive flexibility, inhibition, and planning, as potential risk markers for the development of impulsive behaviors. On a similar wavelength, Zhang et al. (2021) [8] propose that the presence of ICDs is statistically significantly associated with frontal circuit dysfunction, even when controlling for sociodemographic factors such as age, gender, and disease duration.

The assessment of these functions using tools such as the Montreal Cognitive Assessment (MoCA) and the Frontal Assessment Battery (FAB) enables the early detection of dysfunctions in cognitive control and behavioral adjustment in Parkinson’s disease [9,10]. These areas are closely linked to increased vulnerability to the onset of ICDs. These tools combine high sensitivity with easy clinical application, making them suitable for both early screening and longitudinal monitoring. In this context, the current research approach abandons the one-dimensional interpretation of ICDs as a simple side effect of dopaminergic therapy and is now oriented towards understanding them as a multifactorial neurobehavioral phenomenon, in which functional disorders of frontal circuits, pharmacological mechanisms, and individual cognitive characteristics are intertwined.

The association between ICDs and cognitive deficits in Parkinson’s disease has emerged as one of the most critical research issues in recent years. The focus has shifted from a unidimensional pharmacological interpretation to a more complex view, where ICDs are associated with dysfunctions of executive functions and, in particular, with deficits in flexibility, inhibition, and behavioral adaptation to changing demands. Data from numerous studies converge on the view that ICDs are not simply a pharmacogenetic complication of dopaminergic treatment but may arise from or be enhanced by preexisting dysfunctions in higher cognitive functions that regulate self-control and behavioral adaptation. The findings of Santangelo et al. [3] provide compelling evidence for a significant inverse relationship between cognitive performance—assessed through instruments like the MoCA and FAB—and the presence of impulse control disorders in patients with Parkinson’s disease. Importantly, this association appears to persist regardless of variables such as gender, age, or disease duration.

In parallel, the review study by Martini et al. [5] collected data from a wide range of studies that utilized neuropsychological assessment tools, including the Stroop Test and the Wisconsin Card Sorting Test (WCST) [11]. Patients with ICDs exhibited systematically increased errors, limited cognitive flexibility, and reduced ability to inhibit responses, findings suggestive of deficits in executive function rather than generalized cognitive impairment. These findings make it necessary to evaluate higher cognitive processes, such as inhibition, planning, and decision-making, as predictors of ICD behaviors. Incorporating sensitive cognitive screening tools such as the MoCA and FAB into routine clinical assessment enables the early detection of executive impairments, which may either precede or develop alongside pharmacological treatment. Identifying such patterns plays a pivotal role in enabling early clinical response, informing prognosis and guiding personalized treatment planning for individuals with Parkinson’s disease who present with impulse control disorders.

Although pharmacological treatment, in particular dopaminergic agonists, is considered the most documented causal factor in the occurrence of ICDs in patients with Parkinson’s disease, current research evidence demonstrates that specific sociodemographic characteristics moderate the risk of such behaviors. In particular, according to Zhang et al. [8], gender appears to differentiate the type of ICDs developed. Male patients exhibit higher rates of hypersexuality and pathological gambling, while females show an increased frequency of compulsive buying and episodes of overeating. These differences may reflect both biological influences (hormonal factors, dopamine metabolism) and sociocultural conditions that affect the expression of behaviors.

Age also appears to be a modifying factor. The onset of PD at a younger age has been associated with a higher incidence of ICDs [6], possibly due to prolonged exposure to dopaminergic treatment, as well as increased brain plasticity that may enhance the response of the limbic circuitry to reinforcing stimuli. Younger age is also associated with higher levels of mobility and social interaction, which may facilitate the onset of externalizing behaviors.

Finally, the level of education acts as a protective factor. Lower levels of education have been associated with reduced cognitive reserve, specifically a diminished capacity for cognitive control, strategic processing, and behavioral inhibition. Hindle et al. [12] note that lower-educated individuals might struggle more with self-regulation and managing temptations, which can make them more likely to exhibit impulsive compulsive behaviors.

ICDs are a class of psychiatric disorders that have a multilevel impact on patients’ functioning. Studies have demonstrated that patients with ICDs experience increased levels of interpersonal conflict, financial problems, social withdrawal, and psychological distress [2,13]. In addition, comorbidity with depression and anxiety leads to worsening clinical course and increased hospitalizations. In quality of life questionnaires (e.g., PDQ-39), patients with ICDs score consistently lower, particularly in the domains of “emotional well-being” and “social functioning”. This reinforces the need for early detection and intervention, even in the early stages of the disease.

Although there is an expanding body of research on impulse control disorders in Parkinson’s disease, there are still important gaps in our comprehension of the characteristics, mechanisms, and clinical consequences of these behaviors. In particular, there is a strong need for a thorough and systematic examination of the relationship between ICDs and overall cognitive and executive functioning. Although individual studies have identified cognitive deficits in patients with ICDs, the integration of data through reliable, brief, and widely used tools, such as the MoCA and the FAB, remains limited. The use of these tools may allow simultaneous assessment of global cognitive status and frontal executive function. Moreover, the degree to which these results can be applied in clinical settings remains uncertain, particularly regarding the early identification of individuals at high risk for ICDs. At present, ICDs are frequently diagnosed at advanced stages, when they have already generated considerable social, economic, and psychological challenges. The integration of neuropsychological tools (MoCA/FAB) and behavioral assessments (QUIP/QUIP-RS) into clinical practice can help develop early prognostic indicators and facilitate personalized intervention. However, the selection criteria and timing of assessment have not yet been standardized, which makes widespread implementation difficult.

In summary, impulse control disorders in PD are frequent and clinically critical behavioral manifestations, with a strong correlation with dopaminergic pharmacotherapy but also with endogenous neurocognitive and psychosocial factors. Their combined study in the light of general cognitive function (MoCA) and frontal executive performance (FAB) may lead to a more meaningful understanding of the underlying mechanisms and more effective prevention and management. The present study aims to advance the existing field of research by proposing a model that correlates behavioral and cognitive indicators with potential future applications in clinical practice. The primary target of the current study is to investigate the relationship between the presence and intensity of impulsive compulsive behaviors, as captured by the QUIP (Questionnaire for Impulsive-Compulsive Disorders in Parkinson’s Disease), and cognitive function in patients with Parkinson’s Disease. Specifically, we examine whether higher scores on the QUIP correlate with lower performance on global cognitive function, as assessed via the MoCA, and on frontal-executive function, as measured via the FAB. A secondary objective is to assess the frequency and distribution of individual types of behaviors (e.g., pathological gambling, overeating, compulsive buying, and medication overuse).

Based on the above, the central research hypothesis of the study is that higher values in the overall MoCA score will be positively correlated with performance on the FAB test. Higher QUIP total score values will be negatively correlated with performance on the MoCA test. If these hypotheses are confirmed, the study’s results could help pinpoint risk factors for ICDs and facilitate the development of tailored prevention and intervention approaches in the clinical management of PD.

## 2. Materials and Methods

### 2.1. Research Design

The present study follows a cross-sectional design to investigate the relationship between impulsive compulsive behaviors and cognitive performance in patients with Parkinson’s disease. The study was conducted in the outpatient clinic of the 1st Neurology Department at Eginition University Hospital. The research procedure was approved by the relevant Ethics Committee and complied with the principles of the Declaration of Helsinki.

### 2.2. Sample

The sample consisted of 55 patients with a confirmed diagnosis of Parkinson’s disease. Of these, 36 (65.5%) were male and 19 (34.5%) were female. Inclusion criteria included a Parkinson’s disease diagnosis according to the UK brain bank criteria, stable medication dosage and sufficient cognitive ability to understand and complete the questionnaires. Subjects with a history of psychotic disorder, severe dementia, or a diagnosis of another neurodegenerative disease were excluded. Severe dementia was defined as a MoCA score below 16, in accordance with commonly used clinical cut-offs indicating moderate-to-severe cognitive impairment. Patients scoring ≥16 were considered eligible for inclusion, provided they were able to comprehend and complete the study procedures.

### 2.3. Evaluation Tools

Tool selection was based on criteria of validity, sensitivity, reliability, and clinical relevance to the most common behavioral phenomena in Parkinson’s disease. The literature suggests that effective recording of impulse control disorders (ICDs) and cognitive functioning requires a multilevel assessment that includes both self-report questionnaires and cognitive functioning tests. For this purpose, four widely used and documented tools were employed: the QUIP, MoCA, FAB, and GDS.

QUIP (Questionnaire for Impulsive-Compulsive Disorders) in Parkinson’s Disease: The QUIP is the most established screening tool for recording impulsive and compulsive behaviors in patients with PD. It was developed by Weintraub et al. (2009) and has been used in numerous studies and drug trials [14]. It assesses the presence of the most common ICDs associated with AD medication, namely pathological gambling (gambling), hypersexuality, compulsive buying (compulsive buying), episodic binge eating (binge eating), compulsive medication overuse (dopaminergic medication overuse), and obsession with order and organization (punding/organizing behaviors). The tool has been reported to have a sensitivity of between 86% and 97% for detecting ICDs at an early stage. However, specificity may be lower, making it primarily a screening tool rather than a diagnostic tool [14,15].

MoCA (Montreal Cognitive Assessment): The MoCA is an international standard for the brief and comprehensive assessment of global cognitive function. It was created by Nasreddine et al. (2005) [16] and is considered more sensitive than the MMSE for detecting mild cognitive decline, particularly in populations with executive dysfunction, such as those with Parkinson’s disease (PD). It includes 30 items and focuses on the following domains: visuospatial and executive functions, attention and concentration, memory, verbal fluency, abstract thinking, and orientation.

Scores < 26 have been shown to correlate with early cognitive decline, and in patients with PD its association with executive deficits is strong [17]. In the present study, it was used as a global index of cognitive competence correlated with the QUIP and FAB.

FAB (Frontal Assessment Battery): The FAB was developed by Dubois et al. (2000) [18] and assesses individual aspects of frontal and executive functions through six short subtests. These include 1. assessing abstract thinking and the ability to find similarities. 2. Mental Flexibility (verbal fluency): Tests verbal fluency and cognitive fluency. 3. Motor Programming (Luria motor series): Assesses the ability to perform programmed motor sequences. 4. Sensitivity to Interference (Conflicting Instructions): Tests the ability to manage conflicting instructions. 5. Inhibitory Control (Go/No-Go task): Assesses the ability to inhibit automated responses. 6. Environmental Autonomy (prehension behavior): Tests the urge to grasp objects reflexively (grasp reflex). The total score ranges from 0 to 18, with values < 13 being associated with frontal dysfunction. The FAB is widely used to assess executive deficits in PD and is significantly associated with the occurrence of ICDs [19]. In the present study, it was utilized to investigate frontal-executive deficits independent of the general cognitive profile specifically.

GDS (Geriatric Depression Scale—GDS-30): The GDS is a weighted self-report questionnaire for the detection of depressive symptoms in the elderly in general and in patients with neurodegenerative diseases. The 30-question version was used in the present study. It is worth noting that, particularly in patients with Parkinson’s disease, the GDS-30 has been evaluated as a reliable tool for detecting depressive disorder, even in cases of mild depression. According to McDonald et al. (2006) [20], the GDS demonstrates sufficient content and differential diagnostic validity in populations with Parkinson’s disease, enabling its integration into both clinical and research procedures. Its use in the present study allowed us to test the possible influence of mood on cognitive performance and the manifestation of impulsive behaviors. In the context of PD, depression is a common comorbidity and can affect both cognitive performance and behavioral self-regulation [4]. The inclusion of the GDS in the protocol enables the interpretation of associations with the QUIP, MoCA, and FAB while controlling for the effect of mood on other variables.

### 2.4. Data Collection Process

Patients were evaluated in individual sessions, each lasting approximately 30 to 40 min, in a quiet outpatient clinic setting. A qualified healthcare professional administered all instruments in a fixed order of presentation. Written informed consent was obtained from all patients.

### 2.5. Statistical Analysis

Descriptive statistics were calculated for age, years since the diagnosis of PD, and years of education. Additionally, mean scores and standard deviations were computed for the MoCA, FAB, GDS, and QUIP. Pearson correlation coefficients were used to examine the associations between the MoCA and QUIP, as well as between the MoCA and FAB. Statistical significance was set at *p* = 0.05.

## 3. Results

### 3.1. Sample Characteristics

The study sample consisted of 55 patients diagnosed with Parkinson’s disease (PD), assessed at the First Neurological Clinic of Aeginition University Hospital in Athens. As shown in Table 1, the mean age of participants was 62.60 years (*SD* = 13.54). Among the 51 participants with available data on disease duration, the mean time for age onset of Parkinson’s disease was 7.17 years (*SD* = 5.90). The mean level of formal education was 11.94 years (*SD* = 3.00). Regarding sex distribution, 36 participants (65.5%) were male and 19 (34.5%) were female. Handedness was recorded; however, due to limited variability and the absence of any notable pattern in relation to cognitive measures, no further analyses were conducted on this variable.

### 3.2. Descriptive Statistics of Evaluation Tools

Participants completed a neuropsychological battery including the Montreal Cognitive Assessment (MoCA), Frontal Assessment Battery (FAB), Questionnaire for Impulsive-Compulsive Disorders in Parkinson’s Disease (QUIP), and the Geriatric Depression Scale (GDS). Descriptive statistics for each measure are presented below (Table 2).

As presented in Table 2, the mean MoCA score was 24.69 (*SD* = 4.25), with a cut-off value of 26. The mean score on the FAB was 14.70 (*SD* = 2.45), relative to a cut-off of 12. Scores on the QUIP averaged 0.64 (*SD* = 1.05), with 1 used as the threshold for the presence of impulsive compulsive behaviors. Of the total number of patients (*n* = 55), 17 (30.35%) showed impulsive compulsive behaviors. The most commonly reported impulsive compulsive behavior was an excessive preoccupation with hobbies (*n* = 7, 38.9%), followed by a tendency toward gambling (*n* = 6, 33.3%). The mean GDS score was 9.33 (*SD* = 6.97), with 10 considered a standard cut-off for elevated depressive symptoms.

**Figure 1:**
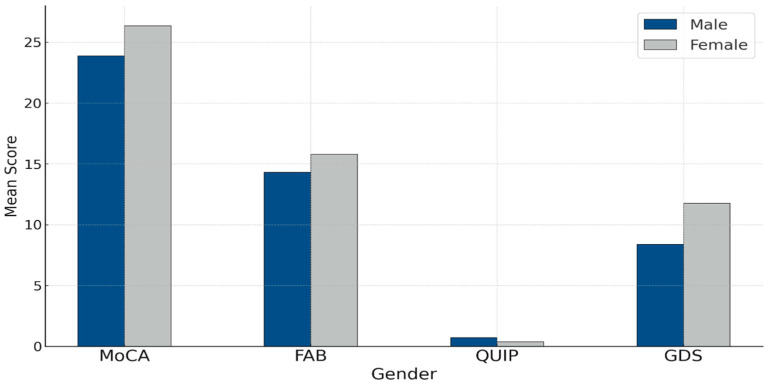
Mean scores in evaluation tools by gender.

Figure 1 presents the mean scores in evaluation tools (MoCA, FAB, GDS, QUIP) across gender groups. Female participants showed slightly higher mean scores on the MoCA and FAB scales, as well as higher scores on the GDS. Males had a slightly higher average on the QUIP scale, reflecting more frequent impulsive compulsive behaviors.

### 3.3. Correlational Analyses

As the sample size was greater than 30, the data were assumed to approximate a normal distribution based on the Central Limit Theorem, supporting the use of Pearson correlation coefficients to explore relationships among the neuropsychological measures. Results are summarized in Table 3.

A statistically significant negative correlation was found between the MoCA and QUIP scores (*r* = –0.291, *p* = 0.038), indicating a weak to moderate inverse relationship between the variables, where lower cognitive scores corresponded to higher impulsive compulsive behaviors. Additionally, there was a statistically significant negative correlation between the QUIP scores and performance on the MoCA attention subtests (Forward Digit Span, Backward Digit Span, and Vigilance tasks), (*r* = –0.389, *p* = 0.009).

A significant positive correlation was observed between the MoCA and FAB scores (*r* = 0.588, *p* = 0.000), reflecting a strong association between global cognitive functioning and executive functioning, as measured by these instruments (Table 3). No significant relationship was found between the QUIP and FAB scores (*r* = –0.179, *p* = 0.213), suggesting that performance on the QUIP was not associated with frontal lobe function as measured by the FAB.

Two linear regression models examined the association between attention scores on the MoCA and impulsive compulsive behaviors measured by the QUIP. In the first model, which included MoCA attention and age, attention scores significantly predicted the QUIP total scores (B = −0.327, SE = 0.145, *p* = 0.030), whereas age was not statistically significant (*p* = 0.319). This model explained 17.2% of the variance in the QUIP scores (R^2^ = 0.172). In the second model, including MoCA attention and Parkinson’s disease duration, attention scores again emerged as a significant predictor of QUIP scores (B = −0.323, SE = 0.145, *p* = 0.032), while disease duration was not significant (*p* = 0.294). This model accounted for 16.6% of the variance (R^2^ = 0.166). Across both models, the association between attention scores and impulsive compulsive symptoms remained statistically significant when each demographic (age) or clinical (PD duration) variable was entered into the model.

**Figure 2:**
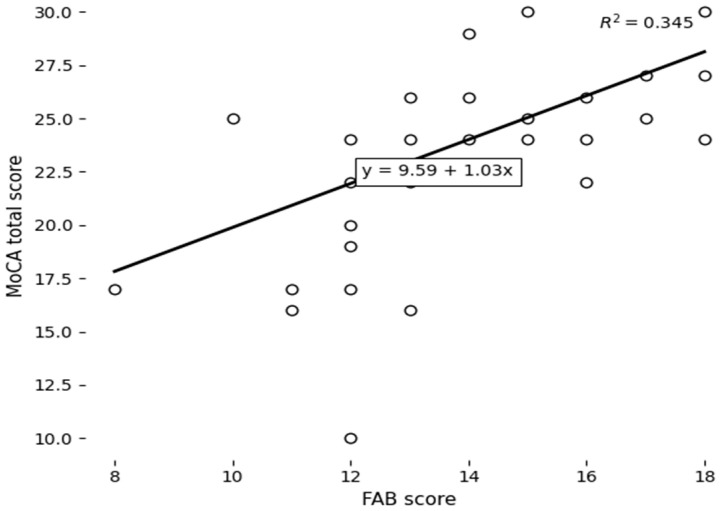
Relationship between FAB and MoCA scores.

A scatterplot was used to explore the relationship between the FAB and MOCA total scores (Figure 2). FAB scores varied from 10 to 18, while MOCA scores varied from 10 to 30. The plot indicates a positive relationship between FAB and MOCA scores, suggesting that higher scores on the FAB are associated with better performance on the MOCA. Participants with lower FAB scores showed a wider spread in MOCA performance, including several individuals with low cognitive scores. On the other hand, participants with higher FAB scores generally achieved higher and more consistent MOCA scores.

Medication effects were examined due to their established relevance to impulsive compulsive behaviors in Parkinson’s disease. The mean total LEDD across all patients (N = 53) was 698.4 mg (*SD* = 452.6), while the mean dopamine agonist LEDD among patients receiving agonists (*n* = 28) was 209.2 mg (*SD* = 169.4); 27 patients were not receiving dopamine agonists. An independent samples t-test showed no significant difference in QUIP total scores between patients receiving dopamine agonists and those not receiving them, t (42.55) = −1.73, *p* = 0.091 (equal variances not assumed). Pearson correlation analyses revealed no significant association between QUIP scores and dopamine agonist LEDD (*r* = 0.137, *p* = 0.337). In contrast, a significant positive correlation was observed between QUIP scores and total LEDD (*r* = 0.413, *p* = 0.003).

## 4. Discussion

Our current research evaluated global cognitive functioning and impulsive compulsive behaviors using the MoCA and QUIP tools, respectively. The goal was to investigate the potential link between impulse control disorders (ICDs) and global cognitive functioning in individuals diagnosed with Parkinson’s disease. The statistical findings indicated a weak to moderate negative correlation between global cognitive performance and impulsive compulsive behaviors (*r* = –0.291, *p* = 0.038). The observed trend is consistent with findings from previous research suggesting that the presence of impulsive behaviors in Parkinson’s patients may be linked to cognitive decline, particularly in areas related to executive function [3]. These findings suggest that reduced global cognitive functioning and behavioral dysregulation may represent parallel or interacting manifestations of underlying neurobiological changes in Parkinson’s disease.

Interestingly, there was a statistically significant negative relationship between QUIP scores and performance on the MoCA attention subtests (Forward Digit Span, Backward Digit Span, and Vigilance tasks), (*r* = –0.389, *p* = 0.009). This finding aligns with prior studies indicating that difficulties in impulse control are commonly associated with impairments in executive functions, particularly attentional processes. However, given the cross-sectional nature of the study, it cannot be determined whether attentional deficits precede, follow, or develop concurrently with impulsive compulsive behaviors [3,8].

Two linear regression models were conducted to examine the link between attention and impulsive compulsive behaviors, each including MoCA attention scores and one additional demographic and clinical variable, age and disease duration. In both models, attention remained a significant predictor of QUIP scores, suggesting this relationship was not fully accounted for by age or disease duration. Adding each variable separately allowed us to examine its effect without compromising model stability given the modest sample size. These results are exploratory and should be interpreted cautiously, until replicated in larger samples.

The significant positive association identified between MoCA and FAB scores in this study underscores the tight relationship between global cognitive efficiency and executive functioning in patients with Parkinson’s disease. This finding is consistent with the existing literature, which suggests that cognitive decline in PD is not limited to isolated memory deficits but often encompasses frontal-executive dysfunctions, particularly in domains such as mental flexibility, abstraction, and inhibitory control [3,17].

However, the absence of a statistically significant correlation between FAB scores and QUIP total scores warrants closer scrutiny. Conceptually, it may appear counterintuitive, given that impulse control disorders (ICDs) are frequently conceptualized as a manifestation of frontal-executive dysfunction. Nevertheless, this lack of association suggests that the executive processes measured by the FAB may not fully capture the cognitive substrates underlying impulsive compulsive behaviors in PD.

A plausible explanation for this dissociation lies in the FAB itself. While the FAB is a validated and efficient tool for assessing broad aspects of frontal lobe functioning, it predominantly evaluates higher-order cognitive control in neutral, non-affective contexts. In contrast, ICDs are fundamentally disorders of reward sensitivity, affective salience, and risk-reward decision-making, which are heavily mediated by ventromedial prefrontal, orbitofrontal, and limbic–striatal circuits, rather than the dorsolateral prefrontal networks primarily assessed by the FAB [8,21].

Moreover, the FAB’s subtests, such as conceptualization, motor programming, and environmental autonomy, are more reflective of structured executive processes. These may not be sufficiently sensitive to capture impulsivity and compulsivity, which are often driven by dysregulated reinforcement learning, impaired delay discounting, and heightened cue-reactivity—cognitive-affective processes that are not adequately probed by bedside tasks.

Another consideration is that the FAB, by design, assesses relatively “cold” executive functions—those devoid of emotional or motivational context. In contrast, ICDs are primarily governed by “hot” executive processes, involving emotionally charged decision-making and reward processing [22]. Neuroimaging studies consistently demonstrate that patients with ICDs exhibit hyperactivation in the mesolimbic dopaminergic pathway, particularly the nucleus accumbens and orbitofrontal cortex, rather than uniform dysfunction across frontal cortical regions [8]. From a clinical perspective, these findings suggest that screening protocols for impulse control disorders in Parkinson’s disease may benefit from combining the FAB with reward-sensitive or affect-laden executive tasks, in order to more effectively capture deficits related to impulsivity and motivational decision-making.

This neurobiological dissociation may explain why the MoCA, as a global cognitive screener encompassing multiple domains—including attention, visuospatial abilities, memory, and executive function—shows a robust association with the FAB. Both instruments reflect general frontal-cortical integrity and overall cognitive status. Conversely, the lack of association between the FAB and QUIP suggests that impulsivity in Parkinson’s disease may be better conceptualized as reflecting domain-specific vulnerabilities in reward-based decision-making and affect-driven cognitive control, which may evolve alongside—but not necessarily as a consequence of—global cognitive decline.

Overall, these findings align with contemporary models suggesting that ICDs in Parkinson’s disease reflect the interplay of multiple factors, including dopaminergic pharmacological effects, altered limbic–striatal signaling, impaired inhibitory control under affective load, and individual differences in reward-based learning systems [10,21]. The current results underscore the need for incorporating more sensitive and ecologically valid neuropsychological tasks, such as the Iowa Gambling Task, Delay Discounting paradigms, or the Cambridge Gamble Task in the assessment of ICD risk in Parkinson’s disease.

The link between social functioning and environmental autonomy in PD may reflect broader evidence that social environments shape brain connectivity and, consequently, behavioral regulation and psychopathology. For instance, in adolescents, prosocial peer networks are associated with increased fronto-striatal and cingulate volumes and more adaptive connectivity patterns, which mediate reduced behavioral problems, whereas exposure to delinquent peers is linked to altered default-mode and fronto-limbic connectivity and greater behavioral dysregulation [23]. Similarly, in PD, reduced environmental autonomy may not only mirror cognitive limitations captured by structured executive tests such as the FAB, but also illustrate how social and environmental contexts interact with neural systems supporting behavioral control. Contemporary models of neurological recovery emphasize dynamic interactions between distributed neural networks rather than isolated regional dysfunction. Evidence from studies on hemispheric interactions and compensatory pathways suggests that preserved or adaptive network connectivity may partially offset focal executive deficits, shaping behavioral outcomes in complex environments. Such compensatory mechanisms have been documented across neurological conditions, highlighting the role of network reorganization in maintaining functional autonomy despite localized impairment [24]. This perspective suggests that patients’ difficulties in regulating behavior—particularly in socially complex or emotionally salient situations—may arise from the interplay between neural substrates and external environments, underscoring the importance of assessing both intrinsic executive function and the broader social context in clinical evaluation.

Medication analyses indicated that QUIP scores were not significantly associated with either the presence or dose of dopamine agonists, whereas a modest but significant association was found with total LEDD. Overall, these results suggest that impulsive compulsive symptoms might be more closely linked to overall dopaminergic medication load (LEDD) than to agonist exposure alone. Although dopamine agonists are commonly implicated in the development of ICDs [6], symptom severity in this sample appeared to reflect total dopaminergic exposure rather than agonist use specifically. These findings emphasize the importance of considering overall dopaminergic load when examining impulsive compulsive behaviors in Parkinson’s disease, particularly in clinical samples, while noting the limitations of a relatively small sample size and potentially restricted range of agonist doses.

At this point we should highlight certain limitations of the present report. The study involved multiple correlational analyses across demographic, cognitive, and behavioral measures (MoCA total and sub-scores, the FAB, GDS, and QUIP), increasing the risk of Type I error. No formal correction for multiple comparisons was applied, give the exploratory nature of the study. The modest sample size (*n* = 55) further limits statistical power, particularly for subgroup analyses, so a lack of significance may reflect limited power rather than absence of effect (e.g., FAB–QUIP correlations). Accordingly, significant results—especially the correlation with the MoCA attention sub-score—should be interpreted cautiously and require replication in larger samples. This approach allows for exploratory investigation while maintaining transparency about potential statistical limitations.

## 5. Conclusions

This study suggests that preliminary cognitive assessments, such as the MoCA, may provide clinically useful information regarding impulsive compulsive behaviors in individuals with Parkinson’s disease. In particular, attention-related subtests showed stronger associations with impulsive behaviors, while the overall MoCA score may still represent a pragmatic tool for preliminary cognitive screening, given the observed associations with ICD symptom severity. These findings may support clinicians in informing clinical monitoring and assessment strategies. However, due to the limited sample size and the cross-sectional design of the study, the results should be interpreted with caution, and no conclusions can be drawn regarding causality or the temporal relationship between cognitive performance and impulse control disorders.

## Figures and Tables

**Table 1:** Descriptive overview of demographic characteristics.

Variable	*n*	Mean (*M*)	Standard Deviation (*SD*)
Age *	50	62.60	13.54
Education *	51	11.94	3.00
Duration of Disease *	51	7.17	5.90

* Age, duration of the disease and education are expressed in years.

**Table 2:** Average total scores for each assessment tool.

Test	*n*	Mean (*M*)	Standard Deviation (*SD*)
MoCA	55	24.69	4.25
FAB	54	14.70	2.45
GDS	51	9.33	6.97
QUIP	51	0.64	1.05

**Table 3:** Pearson correlations of MoCA with FAB and QUIP scores.

Test	*r*	*p* Value
MoCA—QUIP	−0.291	0.038
MoCA/Attention—QUIP	−0.389	0.009
MoCA—FAB	0.588	0.000
FAB—QUIP	−0.179	0.213

## Data Availability

The database will be available following reasonable requests.

## Abbreviations

The following abbreviations are used in this manuscript: PD, Parkinson’s Disease; ICDs, Impulse Control Disorders; MoCA, Montreal Cognitive Assessment; FAB, Frontal Assessment Battery; QUIP, Questionnaire for Impulsive-Compulsive Disorders in Parkinson’s Disease; GDS, Geriatric Depression Scale.
